# Measurement Variability Following MRI System Upgrade

**DOI:** 10.3389/fneur.2019.00726

**Published:** 2019-07-16

**Authors:** Olivier Potvin, April Khademi, Isabelle Chouinard, Farnaz Farokhian, Louis Dieumegarde, Ilana Leppert, Rick Hoge, Maria Natasha Rajah, Pierre Bellec, Simon Duchesne, the CIMA-Q group, the CCNA group

**Affiliations:** ^1^Centre de Recherche CERVO, Quebec, QC, Canada; ^2^Image Analysis in Medicine Lab, Ryerson University, Toronto, ON, Canada; ^3^McGill University, Montreal, QC, Canada; ^4^Montreal Neurological Institute, Montreal, QC, Canada; ^5^Douglas Mental Health University Institute, Montreal, QC, Canada; ^6^Institut Universitaire en Gériatrie de Montréal, Montreal, QC, Canada; ^7^Département de Psychologie, Université de Montréal, Montreal, QC, Canada; ^8^Département de Radiologie et de Médecine Nucléaire, Université Laval, Quebec, QC, Canada

**Keywords:** neuroimaging, magnetic resonance imaging, MRI upgrade, variability, longitudinal studies, morphometry, Siemens healthcare

## Abstract

Major hardware/software changes to MRI platforms, either planned or unplanned, will almost invariably occur in longitudinal studies. Our objective was to assess the resulting variability on relevant imaging measurements in such context, specifically for three Siemens Healthcare Magnetom Trio upgrades to the Prisma^fit^ platform. We report data acquired on three healthy volunteers scanned before and after three different platform upgrades. We assessed differences in image signal [contrast-to-noise ratio (CNR)] on T1-weighted images (T1w) and fluid-attenuated inversion recovery images (FLAIR); brain morphometry on T1w image; and small vessel disease (white matter hyperintensities; WMH) on FLAIR image. Prisma^fit^ upgrade resulted in higher (30%) and more variable neocortical CNR and larger brain volume and thickness mainly in frontal areas. A significant relationship was observed between neocortical CNR and neocortical volume. For FLAIR images, no significant CNR difference was observed, but WMH volumes were significantly smaller (-68%) after Prisma^fit^ upgrade, when compared to results on the Magnetom Trio. Together, these results indicate that Prisma^fit^ upgrade significantly influenced image signal, brain morphometry measures and small vessel diseases measures and that these effects need to be taken into account when analyzing results from any longitudinal study undergoing similar changes.

## Introduction

Magnetic resonance imaging (MRI) is by now of routine use in neuroscience studies of the living human brain. A multiplicity of contrast mechanisms has been devised to provide anatomical, cerebrovascular, functional, pathological, and metabolite information, in neurological and psychiatric diseases alike. Frequently, in order to increase participation and achieve sample sizes of statistical significance, investigators rely on acquisitions performed at multiple centers—and hence, using multiple imaging systems. Unfortunately, given that MRI signals are not recorded in absolute values, different platforms will produce different intensities for a given contrast, based on the physics of acquisition. These intensity differences will lead to between-system contrast differences, which in turn will impact measurements, for example morphometric estimates (1–7). Harmonized protocols, geometric phantom correction and human volunteer calibration are quality control techniques which can be used to reduce these differences, and hence their impact when comparing participant populations between centers.

The situation is otherwise complicated when changes occur within a given center, for example when technical and/or managerial pressure requires preventive (scheduled) or corrective software and especially hardware updates. On the one hand, cross-sectional designs would be mildly affected; one could consider participants scanned pre- and post-upgrades as though they had been seen at two different sites. On the other hand, longitudinal studies risk being affected; disentangling the effect due to the upgrade from that of the phenomena under study becomes intractable.

Very few studies have assessed MRI scanner upgrade effects using short scan intervals. On T1-weighted images (T1w), increased signal-to noise (SNR) and contrast-to-noise (CNR) ratio have been reported on Siemens Symphony software upgrades (8) and altered brain morphometry have been observed following GE Signa software upgrades using a 1-year scan interval (9). Moreover, using the Alzheimer's Disease Neuroimaging Initiative (ADNI) dataset, other authors (10) reported a slight increase in total brain volume following upgraded 1.5T scanners (0.33% GE Signa Excite to Signa HDx and 0.39% Siemens Symphony to Symphony TIM). However, other studies did not observe any significant difference in neocortical thickness and subcortical regions' volume following Siemens Sonata-Avanto and Trio-Tim Trio software/hardware upgrades, within a 6-week scan interval (4, 7).

This situation is common for studies in the context of neurodevelopment or neurodegeneration, where follow-ups tend to last through the expected mid-life update (~3–4 years) if not expected lifetime of a high-caliber research MRI platform (~6–8 years). It was the case for our involvement in both the longitudinal *Quebec Consortium for Early identification of Alzheimer's disease* (the Consortium d'identification précoce de la maladie d'Alzheimer – Québec; CIMA-Q; https://www.cima-q.ca) and the *Canadian consortium for neurodegeneration and aging* (CCNA; https://www.ccna-ccnv.ca). Common to both studies, three Siemens Trio systems have undergone major hardware upgrades (Prisma^fit^) within the span of 1 year, while recruitment and follow-up were undergoing.

Faced with this inevitability, our strategy was to measure the variability induced by the upgrade by measuring changes in pre/post scans of human volunteers for measurements of interest to both studies. We argue that this variability can serve as a threshold against which to compare any future change being detected in the course of longitudinal studies that have included these sites. We therefore report in the following chapters our quantifying of differences in image signal, anatomical information (brain morphometry), and small vessel disease as relevant measurements that exemplify the degree of variability following a major upgrade. To our knowledge, this study is the first to assess the impact of Prisma^fit^ upgrade on image signal and brain morphometry. Since the Siemens Trio scanner is a model widely used in neuroimaging research (e.g., see Potvin et al. (1) for the scanner characteristics of 23 openly accessible datasets), this upgrade will be likely prevalent in future neuroimaging studies, hence the importance to measure its impact on image signal and brain morphometry.

## Materials and Methods

### Participants and Image Acquisition

Three healthy volunteers (all males; age range 43–47 years old) participated in the study, with images acquired at three different sites, each undergoing a complete system overhaul. Specifically, these were: the McConnell Brain Imaging Center (McGill University, Montreal, Canada), the Douglas Mental Health University Institute's Brain Imaging Center (McGill University, Montreal, Canada) and the Unité de Neuroimagerie Fonctionnelle (Université de Montréal, Montreal, Canada). Each site planned and executed a scheduled upgrade of their Magnetom Trio to Prisma^fit^ platforms (Siemens Medical Systems, Erlangen, Germany). These major upgrades involved a complete retrofit of the signal transmission and reception chains, reconstruction hardware, and coils.

To test pre-post upgrade changes, the three volunteers were scanned three times before and twice after at McConnell Brain Imaging Center (BIC). One of these participants was scanned five times before and five times after at the Unité de Neuroimagerie Fonctionnelle (UNF); and twice before and once after at the Institut en santé mentale de l'hôpital Douglas (ISMD). Altogether, there were 16 scans acquired before and 12 scans after the upgrade. The scan interval ranged between 86 to 150 days (mean: 133.8, std: 26.8).

Part of the data used in this article were obtained from the Consortium pour l'identification précoce de la maladie Alzheimer - Québec (CIMA-Q), founded in 2013 with a $2,500,000 grant from the *Fonds d'Innovation Pfizer - Fond de Recherche Québec – Santé sur la maladie d'Alzheimer et les maladies apparentées*. The main objective was to build a cohort of participants characterized in terms of cognition, neuroimaging and clinical outcomes in order to acquire biological samples allowing (1) to establish early diagnoses of Alzheimer's disease, (2) to provide a well-characterized cohort, and (3) to identify new therapeutic targets. The principal investigator and director of CIMA-Q is Dr. Sylvie Belleville from the Centre de recherche de l'Institut universitaire de gériatrie de Montréal, CIUSSS Centre-sud-de-l'Île-de-Montréal. CIMA-Q represent a common effort of several researchers from Québec affiliated to Université Laval, Université McGill, Université de Montréal, et Université de Sherbrooke. CIMA-Q recruited 290 cognitively healthy participants, with subjective cognitive impairment, mild cognitive impairment, or Alzheimer's disease, between 2013 and 2016.

### Image Acquisition Protocol

All acquisitions were performed following the *Canadian Dementia Imaging Protocol* (11) (www.cdip-pcid.ca), and consisted in (a) a sagittal 3D isotropic T1-weighted (T1w) scan with 1.0 X 1.0 X 1.0 mm^3^ resolution, 256 X 256 matrix, 192 slices, field of view (FOV) of 256 X 256 mm, repetition time (TR) of 2300 msec, echo time (TE) of 2.98 msec, no inversion time (TI), flip angle of 125, and acceleration factor of 2 (Siemens: MP- RAGE-PAT); and (b) an axial fluid attenuated inversion recovery (FLAIR) with resolution 0.9 X 0.9 X 3 mm^3^, fat saturation, 256 X 256 matrix, 48 slices, FOV of 240 X 240 mm, TE of 123 msec, TI of 2500 msec, flip angle of 165, and an acceleration factor of 2. Two sites (BIC and UNF) used the *32-Channel Head Coil* for the pre- and post-upgrade acquisitions, while the ISMD used the *Head Matrix Coil* (12 channels) for the pre- upgrade acquisition and the *Head/Neck 20* coil (20 channels) for the post-upgrade acquisition. This report focuses on the impact of Prisma^fit^ upgrade on measurements of T1w and FLAIR image, but the CDIP also included other sequences (diffusion-tensor (DWI), and resting state functional T2^*^-weighted blood-oxygen-level-dependent sensitive sequence (rsfMRI) images). A separated study is planned for DWI and rsfMRI.

### Image Processing

T1w images were processed using *FreeSurfer* 5.3 (http://freesurfer.net) with default pipeline (recon-all -all) without any flag option. The technical details of these procedures are described in prior publications (12, 13).

### Signal Analysis Measurements

Contrast-to-noise ratio (CNR) was assessed using intensities values with the following formula, using voxel intensities from T1w images with the mean and variance (squared standard deviation) of gray matter (GM) and cerebral white matter (WM):

(1) $$\begin{array}{r} CNR=\frac{{\left(GM\;mean\;-\;WM\;mean\right)}^{2}}{\left(GM\;variance\;+\;WM\;variance\right)} \end{array}$$

where subcortical and neocortical GM and WM volumes were obtained from the aparc and aseg labels generated by *FreeSurfer*. We computed the CNR before (orig.mgz) and after (nu.mgz) inhomogeneity correction. In order to compute CNR for FLAIR images, the same equation as T1w was used. However, since there is little contrast between WM and GM classes in FLAIR, the first tissue class is called the brain class (which is GM + WM as a whole), and the second tissue class is the CSF. In order to compute the mean and standard deviations for the brain and CSF tissues, segmentations of these tissues were required, which was generated using a standardization and segmentation framework for FLAIR MRI (14–16). To ensure that the tissue classes contained pure tissues only, 50% of the middle slices were retained for CNR calculation, ensuring that if the brain extraction algorithm missed any skull at the top or bottom, it would not bias the approach.

### Brain Morphometry Measurements

From *FreeSurfer*, the default subcortical (10 regions per hemisphere) (17) and Desikan-Killiany-Tourville neocortical atlases (31 neocortical regions per hemisphere) volumes and thicknesses were used (18), resulting in 144 regional morphometric measures. Total white matter was defined as the difference between the total brain volume without ventricles and the total gray matter.

### White Matter Hyperintensities Measurement

WMH on FLAIR were assessed using Schmidt et al.'s automated LST toolbox (19), including both T1w and FLAIR images as input.

### Statistical Analyses

For analyses, variables were transformed into comparable scales between participants. For each participant, all values were converted into percentage of its mean. To verify the effect of Prisma^fit^ upgrade on CNR and morphometric measures, we fitted a linear-mixed model for each measure with Prisma^fit^ upgrade as between-factor and subject as a repeated factor with random intercepts for each subject. This model allows to partition out variability due to individual differences and test whether the variance due to the scanner upgrade was higher than error variance which encompasses unknown factors' influence such as noise. Furthermore, we used the Levene's test, which assesses the homogeneity of variances (20), to test differences in variance before and after upgrade. For the regional morphometric measurements (144 measures), we used false discovery rate (FDR) *p*-value correction to adjust for multiple comparisons (21).

Moreover, in order to verify whether signal changes influenced morphometric results, we conducted Pearson's correlation were conducted between CNR and cortical/subcortical volumes. Furthermore, to assess the pre/post upgrade reliability, we computed intraclass correlations (ICC) two-way random effects with multiple measurements (22) with pre and post measurements each treated as a rater. To assess intra-scanner morphometric reliability, the same ICC was computed, but for pre and post images separately with each measurement treated as a rater.

Using 28 images (16 scans acquired before and 12 scans acquired after the upgrade), we calculated that with a two-tailed alpha of 5% and a power of 80%, medium effect sizes (0.55) could be detected (23).

All statistical analyses were conducted in Python using SciPy (24) and StatsModels (25) modules, except ICC which were computed through the ICC function of the psych R package (26) and interpretation were made based on Cicchetti's guidelines (27).

## Results

### Signal Changes Following Upgrade

**Figure 1:**
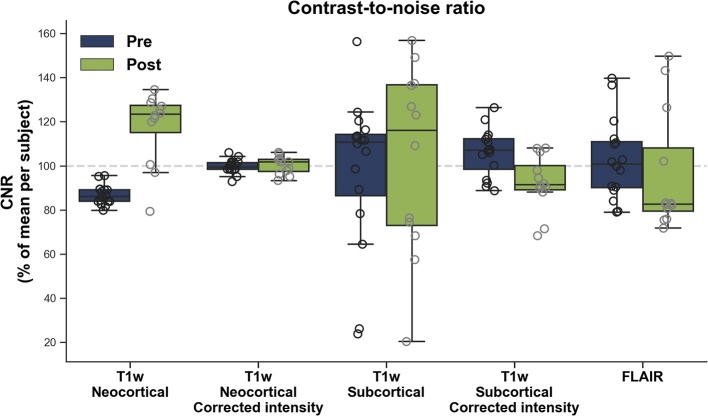
Contrast-to-noise ratio (CNR) before (Pre) and after (Post) Prisma^fit^ upgrade from T1w images and FLAIR images.

Figure 1 shows the CNR before and after upgrade for neocortical and subcortical areas. The Prisma^fit^ upgraded platforms significantly increased neocortical (model estimate change: 30.0%, *p* < 0.0001; mean ± sd: pre = 86.8% ±4.3, post = 117.6% ± 15.8), but not subcortical CNR (1.4%, *p* = 0.8962; pre = 97.8% ± 33.8, post = 103.0% ± 40.8), compared to their Trio counterparts. In addition, Prisma upgrade resulted in higher variability of neocortical (*p* = 0.0459), but not subcortical (*p* = 0.2557) CNR compared to the Magnetom Trio.

After N3 inhomogeneity correction (28), a significant difference in CNR was observed after Prisma^fit^ upgrade for subcortical (-13.8%, *p* = 0.0017; pre = 105.9 ± 10.4, post = 92.1 ± 12.1), but not neocortical (0.3%, *p* = 0.8300; pre = 99.7 ± 3.1, post = 100.4 ± 3.9) areas. The upgrade did not significantly impact the variability of neocortical (*p* = 0.4106) or subcortical (*p* = 0.9021) CNR compared to the Magnetom Trio.

Figure 1 displays the CNR for FLAIR images. No significant differences were observed before and after Prisma^fit^ upgrade in terms of mean (−6.4%, *p* = 0.4613; pre = 102.8% ± 17.7, post = 96.3 ± 26.5) or variance (*p* = 0.5409) of FLAIR CNR.

ICCs revealed that CNR reliability between pre and post Prisma^fit^ upgrade ranged between poor and fair (T1w cortex: 0.04 ± 95CI: −0.13–0.33, with corrected intensities: 0.52 ± −0.34–0.83; T1w subcortical: 0.38 ± −0.74–0.78; with corrected intensities: 0.00 ± 0–0.66–0.53; FLAIR: 0.46 ± −0.46–0.81).

### Brain Morphometry Changes Following Upgrade

**Figure 2:**
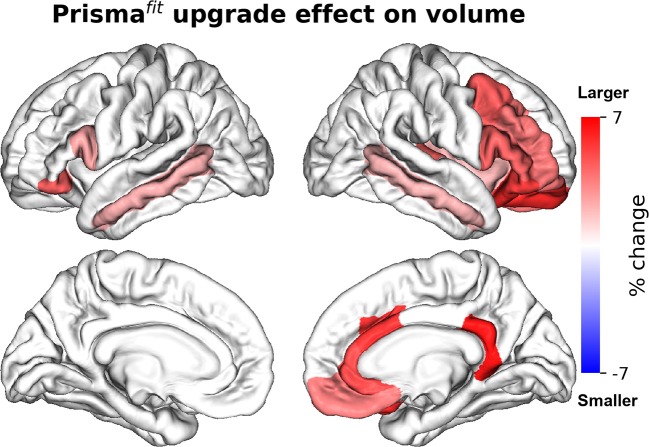
Percentage of change in neocortical regions' volume after Prisma^fit^ upgrade. Only regions that were significant after false-discovery rate correction (*p* < 0.05) are showed.

**Figure 3:**
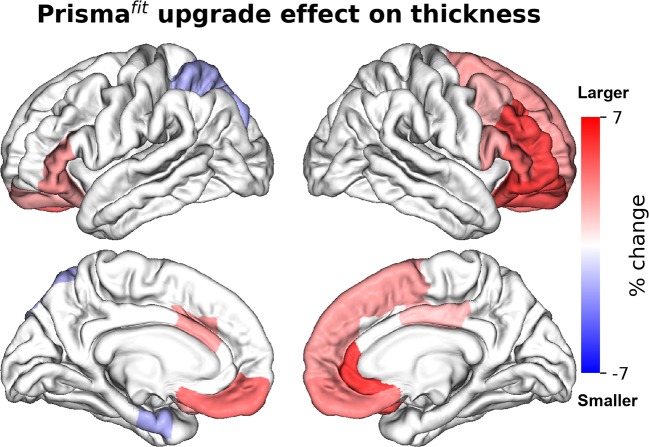
Percentage of change in neocortical regions' thickness after Prisma^fit^ upgrade. Only regions that were significant after false-discovery rate correction (*p* < 0.05) are showed.

**Figure 4:**
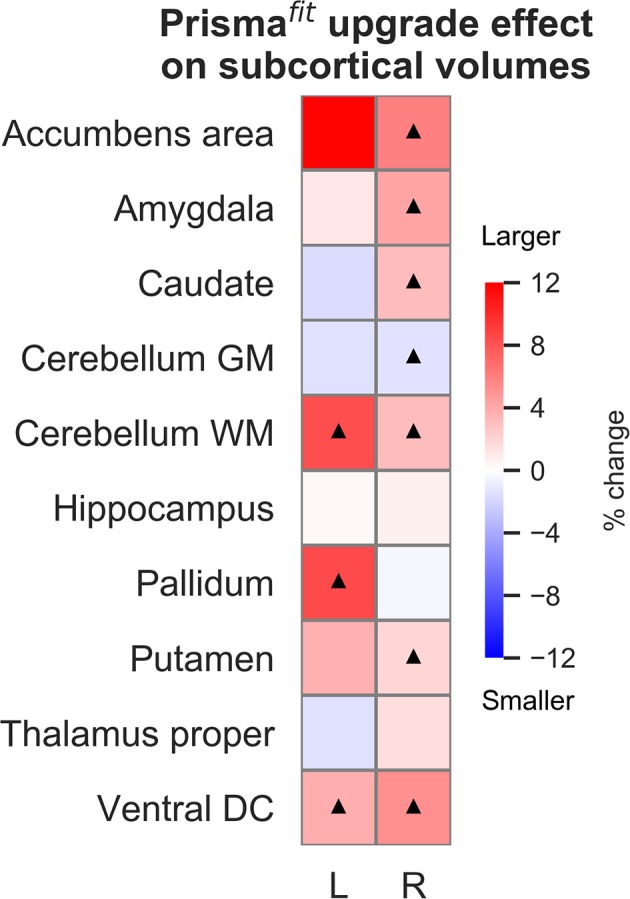
Percentage of change in subcortical regions' volume after Prisma^fit^ upgrade. Black triangles denote regions that were significant after false-discovery rate correction (*p* < 0.05).

Figures 2, 3 show the percentage of change in volume and thickness, respectively, of neocortical regions after the Prisma^fit^ upgrade. Thirty-two neocortical measures (16 volumes and 15 thicknesses) were significantly larger after the upgrade with ranges between 1.9 to 6.4% for volumes and 1.9 to 5.3% for thickness. In addition, two regions were significantly thinner (left entorhinal: −2% and left superior parietal: −2%). Figure 4 displays the percentage of change in volume of subcortical regions after the Prisma^fit^ upgrade. Nine regions had significantly larger volumes (range: 1.9–8.5%) while the right cerebellum cortex had significantly smaller volume after the upgrade.

In terms of variances, all morphometric measures (neocortical volumes, neocortical thicknesses, and subcortical volumes) did not significantly differ between pre and post Prisma^fit^ upgrade after FDR correction (Supplementary Table 1).

**Figure 5:**
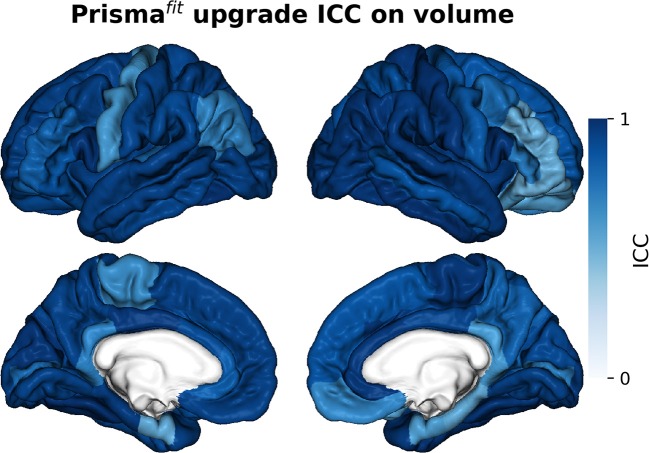
Intraclass correlations (ICC) of neocortical regions' volume before and after Prisma^fit^ upgrade.

**Figure 6:**
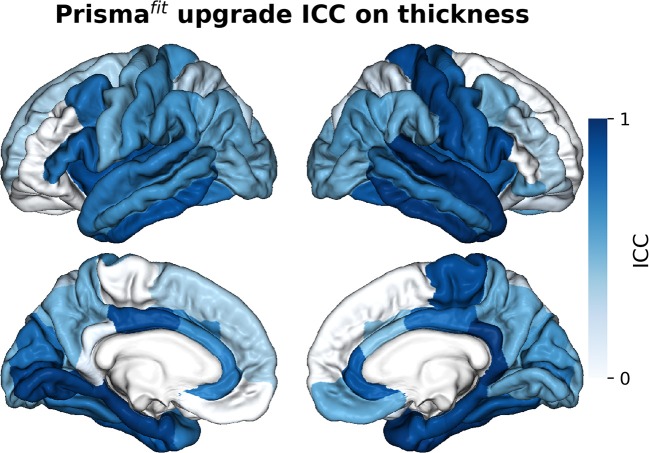
Intraclass correlations (ICC) of neocortical regions' thickness before and after Prisma^fit^ upgrade.

**Figure 7:**
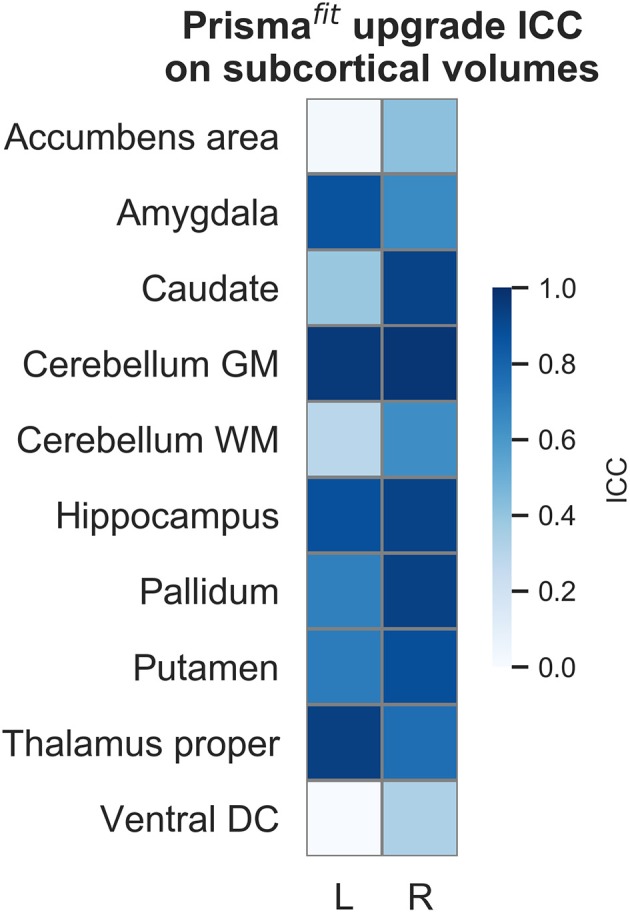
Intraclass correlations (ICC) of subcortical regions' volume before and after Prisma^fit^ upgrade.

Figures 5–7 show ICCs for neocortical volume, neocortical thickness and subcortical volume, respectively. Neocortical regions' volume ICCs were generally excellent (mean: 0.85 ± sd: 0.14) while those for thickness were more variable ranging from poor to excellent (0.60 ±0.30). As could be expected, brain regions showing the largest thickness differences after the upgrade (i.e., frontal areas) displayed the lowest ICCs. Subcortical regions' volume ICCs were mostly good (0.66 ± 0.31), except for the accumbens area, ventral diencephalon, left caudate, and left cerebellum WM, which had poor values.

### Correlation Between CNR and Morphometry

A significant positive correlation was observed between cortical CNR and cortical volume (*r*: 0.623, *p* = 0.0004). After inhomogeneity correction, this relationship was lower, but still significant (*r*: 0.491, *p* = 0.0080). A significant negative correlation was also observed between subcortical CNR and subcortical volume before (*r*: −0.534, *p* = 0.0034), but not after inhomogeneity correction (before *r*: −0.332, *p* = 0.0840).

### WMH Changes Following Upgrade

**Figure 8:**
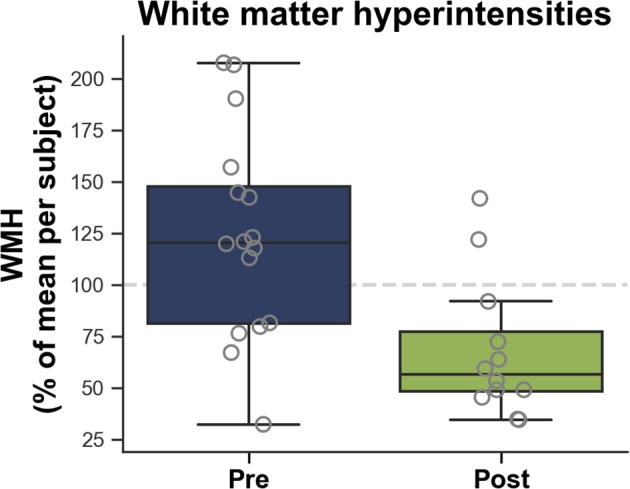
White matter hyperintensities (WMH) measured on FLAIR images according to before (Pre) and after (Post) Prisma^fit^ upgrade.

Figure 8 illustrates that WMH were significantly smaller after Prisma^fit^ upgrade compared to Magnetom Trio acquired FLAIR images (−68%, *p* = 0.0011; pre = 123.8 ± 48.6, post = 68.3 ± 32.5), but no significant difference in terms of variability were observed (*p* = 0.2260). ICC revealed that WMH reliability was fair between pre and post Prisma^fit^ upgrade (0.41).

### Intra-Scanner Morphometric Reliability

Supplementary Figures 1, 2 show the pre and post upgrade ICCs for neocortical volume and thickness, and subcortical volumes, respectively. Reliability was excellent for nearly all regions both pre- (0.92 ± 0.19) and post- (0.90 ± 0.16) upgrade. Only four pre-upgrade measures (right pars triangularis thickness, right superior parietal thickness, left ventral diencephalon, and left isthmus cingulate thickness) and four post-upgrade measures (left isthmus cingulate volume, left paracentral thickness, right lingual thickness, right accumbens area, left medial orbitofrontal thickness) had poor ICCs. Furthermore, WMH reliability was excellent pre- (0.78) and post- (0.88) upgrade.

## Discussion

We aimed to assess the impact of major MRI system upgrades on relevant signal, brain morphometry, and small vessel disease measurements. Despite good reliability within scanner for the Magnetom Trio and Prisma^fit^ when assess separately, we observed that the upgrade generated notable changes on the order of 30% for neocortical CNR; larger morphometric measures up to 6.4% for neocortical regions' volume, 5.3% for neocortical regions' thickness, 8.5% for subcortical regions' volume; and smaller WMH volume of 68%. Such changes are not to be expected of healthy volunteers (all cognitively intact, and aged <50 years old) within such short scan intervals (between 3–5 months). These changes appear consistent with previous results showing increase CNR (8) and larger brain volumes after scanner upgrade (10).

These changes are not negligible and must be taken into consideration when analyzing data; they should be thought of as “floor” values for effect sizes whenever interpreting results coming from systems that have been upgraded, in a longitudinal setting. To wit, these values must be compared to atrophy rates reported for aging (0.83%/year) (29) and Alzheimer's disease (1.9%/year) (30), that are lower than some of the observed upgrade effects. However, in opposition to these multiple significant mean effects, we did not observe any significant heterogeneity of the variance between pre and post upgrade and therefore cross-sectional estimates using either platform are perfectly sound.

This report focuses on the impact of Prisma^fit^ upgrade on measurements of T1w and FLAIR image, but it is likely that similar variability will affect other sequences e.g., diffusion-tensor functional T2^*^-weighted blood-oxygen-level-dependent sensitive sequences. A separate study is planned for these acquisitions, as obtained using the CDIP protocol.

Investigators analyzing data from studies using these platforms will of course want to take particular note of the above results. While we acknowledge that Prisma^fit^ upgrade has notable effects, there is no easy solution to counter these effects in multicentric studies. A correction factor would be hazardous to build since the effects that we observed are highly non-linear, driven by MR physics and systems engineering, and not driven by the participants.

Our results were acquired at three different sites undergoing the same type of upgrade. It is likely that they are therefore representative of the variability for other sites undergoing a similar change, should the investigators have not captured pre/post-upgrade data. However, this is only one of the many systems in operation and hence, these results cannot be assumed to apply for any other configuration. What should be assumed however is that any upgrade will generate variability, even if its magnitude is less than that reported here. It is therefore highly recommended that investigators collect pre/post upgrade data, preferably on a number of volunteers, and test measurements of interest. If this proves impossible, then comparison to other sites undergoing similar changes may pose as a substitute.

## Data Availability

Part of the data used in this article were obtained from the Consortium pour l'identification précoce de la maladie Alzheimer - Québec (CIMA-Q; cima-q.ca).

## Ethics Statement

Ethics approval was obtained from the Institutional Review Board of the Institut universitaire de gériatrie de Montréal. Written informed consent was obtained from all participants.

## Author Contributions

OP: guarantor of integrity of entire study. OP and SD: literature research. OP: statistical analysis. All authors: study concepts/study design, data acquisition, data analysis/interpretation, manuscript drafting, manuscript revision for important intellectual content, approval of final version of submitted manuscript, and manuscript editing.

### Conflict of Interest Statement

Part of the data used in this article were obtained from the Consortium pour l'identification précoce de la maladie Alzheimer - Québec (CIMA-Q; cima-q.ca). As such, the investigators within the CIMA-Q contributed to the design, the implementation, the acquisition of clinical, cognitive, and neuroimaging data and biological samples. A list of the CIMA-Q investigators is available on cima-q.ca. The authors declare that the research was conducted in the absence of any commercial or financial relationships that could be construed as a potential conflict of interest.
