# Supplementary material for: Measurement Variability Following MRI System Upgrade
Source: Front Neurol. 2019 Jul 16;10:726. doi: 10.3389/fneur.2019.00726 (PMC6648007; doi:10.3389/fneur.2019.00726)
Supplement: Supplementary file 1 [file Table_1.DOCX]

Supplementary Table 1. Homogeneity of variances following Prisma fit upgrade for neocortical volume and thickness, and subcortical volume.

| **Measure** | **FDR corrected p value** | **Levene** | **uncorrected p value** |
| --- | --- | --- | --- |
| Pericalcarine rv | 0.9998 | 7.2344 | 0.0123 |
| Superiorparietal rt | 0.9998 | 5.1629 | 0.0316 |
| Inferiorparietal rt | 0.9998 | 5.1584 | 0.0316 |
| Pericalcarine rt | 0.9998 | 4.0850 | 0.0537 |
| Superiorparietal lt | 0.9998 | 3.6909 | 0.0657 |
| Inferiorparietal lt | 0.9998 | 3.5427 | 0.0710 |
| Parsorbitalis rv | 0.9998 | 3.4301 | 0.0754 |
| Left pallidum | 0.9998 | 3.2444 | 0.0833 |
| Supramarginal rt | 0.9998 | 3.1243 | 0.0889 |
| Insula lt | 0.9998 | 3.0381 | 0.0931 |
| Lateraloccipital lt | 0.9998 | 2.5460 | 0.1227 |
| Parsorbitalis rt | 0.9998 | 2.2331 | 0.1471 |
| Rostralmiddlefrontal lt | 0.9998 | 2.0440 | 0.1647 |
| Precentral lv | 0.9998 | 1.8610 | 0.1842 |
| Left accumbens area | 0.9998 | 1.8275 | 0.1881 |
| Right accumbens area | 0.9998 | 1.7682 | 0.1952 |
| Insula rt | 0.9998 | 1.5463 | 0.2248 |
| Lingual rt | 0.9998 | 1.4980 | 0.2320 |
| Cuneus rt | 0.9998 | 1.4330 | 0.2421 |
| Medialorbitofrontal rt | 0.9998 | 1.3911 | 0.2489 |
| Right amygdala | 0.9998 | 1.2010 | 0.2832 |
| Rostralmiddlefrontal rt | 0.9998 | 1.1846 | 0.2864 |
| Lateralorbitofrontal lt | 0.9998 | 1.1522 | 0.2929 |
| Fusiform lt | 0.9998 | 1.0152 | 0.3229 |
| Pericalcarine lv | 0.9998 | 0.9509 | 0.3385 |
| Superiorfrontal rt | 0.9998 | 0.8977 | 0.3521 |
| Precentral lt | 0.9998 | 0.8444 | 0.3666 |
| Left cerebellum white matter | 0.9998 | 0.8356 | 0.3691 |
| Postcentral lt | 0.9998 | 0.8294 | 0.3708 |
| Superiorfrontal lt | 0.9998 | 0.8192 | 0.3737 |
| Rostralanteriorcingulate lt | 0.9998 | 0.7656 | 0.3896 |
| Fusiform rt | 0.9998 | 0.7414 | 0.3971 |
| Left caudate | 0.9998 | 0.7063 | 0.4083 |
| Inferiorparietal lv | 0.9998 | 0.6886 | 0.4142 |
| Right putamen | 0.9998 | 0.6543 | 0.4259 |
| Rostralmiddlefrontal lv | 0.9998 | 0.6432 | 0.4298 |
| Rostralmiddlefrontal rv | 0.9998 | 0.6412 | 0.4305 |
| Transversetemporal lv | 0.9998 | 0.6224 | 0.4373 |
| Caudalanteriorcingulate lt | 0.9998 | 0.5686 | 0.4576 |
| Supramarginal lt | 0.9998 | 0.5573 | 0.4620 |
| Caudalmiddlefrontal rt | 0.9998 | 0.4976 | 0.4868 |
| Parahippocampal rv | 0.9998 | 0.4663 | 0.5007 |
| Paracentral rt | 0.9998 | 0.4576 | 0.5047 |
| Rostralanteriorcingulate rt | 0.9998 | 0.4046 | 0.5303 |
| Pericalcarine lt | 0.9998 | 0.3802 | 0.5429 |
| Lateraloccipital rt | 0.9998 | 0.3660 | 0.5505 |
| Precentral rv | 0.9998 | 0.3627 | 0.5522 |
| Isthmuscingulate lv | 0.9998 | 0.3625 | 0.5523 |
| Right hippocampus | 0.9998 | 0.3499 | 0.5593 |
| Left thalamus proper | 0.9998 | 0.3478 | 0.5604 |
| Lingual rv | 0.9998 | 0.3387 | 0.5656 |
| Left ventraldc | 0.9998 | 0.3330 | 0.5689 |
| Paracentral rv | 0.9998 | 0.3289 | 0.5712 |
| Precuneus rv | 0.9998 | 0.2967 | 0.5906 |
| Isthmuscingulate rv | 0.9998 | 0.2908 | 0.5943 |
| Cuneus lv | 0.9998 | 0.2741 | 0.6050 |
| Entorhinal rv | 0.9998 | 0.2678 | 0.6092 |
| Parsopercularis rt | 0.9998 | 0.2646 | 0.6113 |
| Parsopercularis lt | 0.9998 | 0.2436 | 0.6258 |
| Parahippocampal rt | 0.9998 | 0.2338 | 0.6328 |
| Precentral rt | 0.9998 | 0.2314 | 0.6345 |
| Cuneus lt | 0.9998 | 0.2212 | 0.6421 |
| Transversetemporal rv | 0.9998 | 0.2149 | 0.6468 |
| Parstriangularis lv | 0.9998 | 0.2133 | 0.6480 |
| Superiortemporal rv | 0.9998 | 0.2087 | 0.6516 |
| Inferiortemporal lt | 0.9998 | 0.2024 | 0.6565 |
| Lateralorbitofrontal lv | 0.9998 | 0.1972 | 0.6607 |
| Middletemporal lt | 0.9998 | 0.1960 | 0.6616 |
| Right cerebellum white matter | 0.9998 | 0.1956 | 0.6619 |
| Posteriorcingulate lv | 0.9998 | 0.1948 | 0.6626 |
| Transversetemporal rt | 0.9998 | 0.1843 | 0.6712 |
| Medialorbitofrontal lt | 0.9998 | 0.1804 | 0.6745 |
| Cuneus rv | 0.9998 | 0.1665 | 0.6866 |
| Caudalmiddlefrontal rv | 0.9998 | 0.1574 | 0.6948 |
| Rostralanteriorcingulate rv | 0.9998 | 0.1533 | 0.6986 |
| Lateraloccipital lv | 0.9998 | 0.1493 | 0.7024 |
| Lingual lt | 0.9998 | 0.1461 | 0.7054 |
| Inferiorparietal rv | 0.9998 | 0.1327 | 0.7186 |
| Left hippocampus | 0.9998 | 0.1318 | 0.7195 |
| Superiortemporal lv | 0.9998 | 0.1262 | 0.7253 |
| Caudalmiddlefrontal lv | 0.9998 | 0.1205 | 0.7313 |
| Rostralanteriorcingulate lv | 0.9998 | 0.1186 | 0.7333 |
| Postcentral rt | 0.9998 | 0.1169 | 0.7352 |
| Parsopercularis rv | 0.9998 | 0.1162 | 0.7359 |
| Transversetemporal lt | 0.9998 | 0.1101 | 0.7427 |
| Right caudate | 0.9998 | 0.1032 | 0.7506 |
| Parsorbitalis lv | 0.9998 | 0.0992 | 0.7553 |
| Middletemporal lv | 0.9998 | 0.0915 | 0.7646 |
| Middletemporal rv | 0.9998 | 0.0881 | 0.7690 |
| Superiortemporal rt | 0.9998 | 0.0875 | 0.7697 |
| Posteriorcingulate lt | 0.9998 | 0.0857 | 0.7721 |
| Precuneus lt | 0.9998 | 0.0852 | 0.7727 |
| Parsorbitalis lt | 0.9998 | 0.0845 | 0.7736 |
| Postcentral rv | 0.9998 | 0.0814 | 0.7776 |
| Right ventraldc | 0.9998 | 0.0808 | 0.7785 |
| Caudalanteriorcingulate rv | 0.9998 | 0.0784 | 0.7818 |
| Fusiform rv | 0.9998 | 0.0766 | 0.7842 |
| Superiorparietal lv | 0.9998 | 0.0710 | 0.7920 |
| Lateralorbitofrontal rt | 0.9998 | 0.0637 | 0.8028 |
| Parstriangularis lt | 0.9998 | 0.0628 | 0.8040 |
| Insula rv | 0.9998 | 0.0600 | 0.8083 |
| Superiorparietal rv | 0.9998 | 0.0567 | 0.8136 |
| Lateralorbitofrontal rv | 0.9998 | 0.0518 | 0.8217 |
| Inferiortemporal rv | 0.9998 | 0.0503 | 0.8244 |
| Superiortemporal lt | 0.9998 | 0.0491 | 0.8264 |
| Parstriangularis rt | 0.9998 | 0.0474 | 0.8294 |
| Parahippocampal lt | 0.9998 | 0.0459 | 0.8321 |
| Supramarginal rv | 0.9998 | 0.0433 | 0.8367 |
| Paracentral lv | 0.9998 | 0.0416 | 0.8400 |
| Entorhinal lt | 0.9998 | 0.0412 | 0.8408 |
| Lateraloccipital rv | 0.9998 | 0.0379 | 0.8471 |
| Entorhinal rt | 0.9998 | 0.0354 | 0.8523 |
| Superiorfrontal rv | 0.9998 | 0.0330 | 0.8573 |
| Caudalmiddlefrontal lt | 0.9998 | 0.0278 | 0.8689 |
| Insula lv | 0.9998 | 0.0207 | 0.8868 |
| Left amygdala | 0.9998 | 0.0180 | 0.8942 |
| Supramarginal lv | 0.9998 | 0.0174 | 0.8962 |
| Entorhinal lv | 0.9998 | 0.0138 | 0.9074 |
| Left cerebellum cortex | 0.9998 | 0.0117 | 0.9145 |
| Superiorfrontal lv | 0.9998 | 0.0112 | 0.9166 |
| Left putamen | 0.9998 | 0.0111 | 0.9169 |
| Inferiortemporal rt | 0.9998 | 0.0109 | 0.9178 |
| Middletemporal rt | 0.9998 | 0.0105 | 0.9193 |
| Fusiform lv | 0.9998 | 0.0095 | 0.9233 |
| Right thalamus proper | 0.9998 | 0.0066 | 0.9359 |
| Postcentral lv | 0.9998 | 0.0061 | 0.9383 |
| Posteriorcingulate rv | 0.9998 | 0.0047 | 0.9457 |
| Medialorbitofrontal rv | 0.9998 | 0.0041 | 0.9492 |
| Isthmuscingulate lt | 0.9998 | 0.0041 | 0.9493 |
| Parstriangularis rv | 0.9998 | 0.0040 | 0.9500 |
| Parsopercularis lv | 0.9998 | 0.0026 | 0.9596 |
| Parahippocampal lv | 0.9998 | 0.0025 | 0.9605 |
| Isthmuscingulate rt | 0.9998 | 0.0021 | 0.9637 |
| Caudalanteriorcingulate rt | 0.9998 | 0.0016 | 0.9683 |
| Medialorbitofrontal lv | 0.9998 | 0.0011 | 0.9738 |
| Lingual lv | 0.9998 | 0.0010 | 0.9746 |
| Precuneus rt | 0.9998 | 0.0010 | 0.9755 |
| Paracentral lt | 0.9998 | 0.0007 | 0.9794 |
| Right cerebellum cortex | 0.9998 | 0.0007 | 0.9795 |
| Precuneus lv | 0.9998 | 0.0007 | 0.9798 |
| Posteriorcingulate rt | 0.9998 | 0.0004 | 0.9849 |
| Inferiortemporal lv | 0.9998 | 0.0001 | 0.9924 |
| Caudalanteriorcingulate lv | 0.9998 | 0.0001 | 0.9929 |
| Right pallidum | 0.9998 | 0.0000 | 0.9998 |
| FDR: false-discovery rate. l: left. r: right. v: volume. t: thickness. | | | |
