# Supplementary figures and images for: Measurement Variability Following MRI System Upgrade
Source: Front Neurol. 2019 Jul 16;10:726. doi: 10.3389/fneur.2019.00726 (PMC6648007; doi:10.3389/fneur.2019.00726)

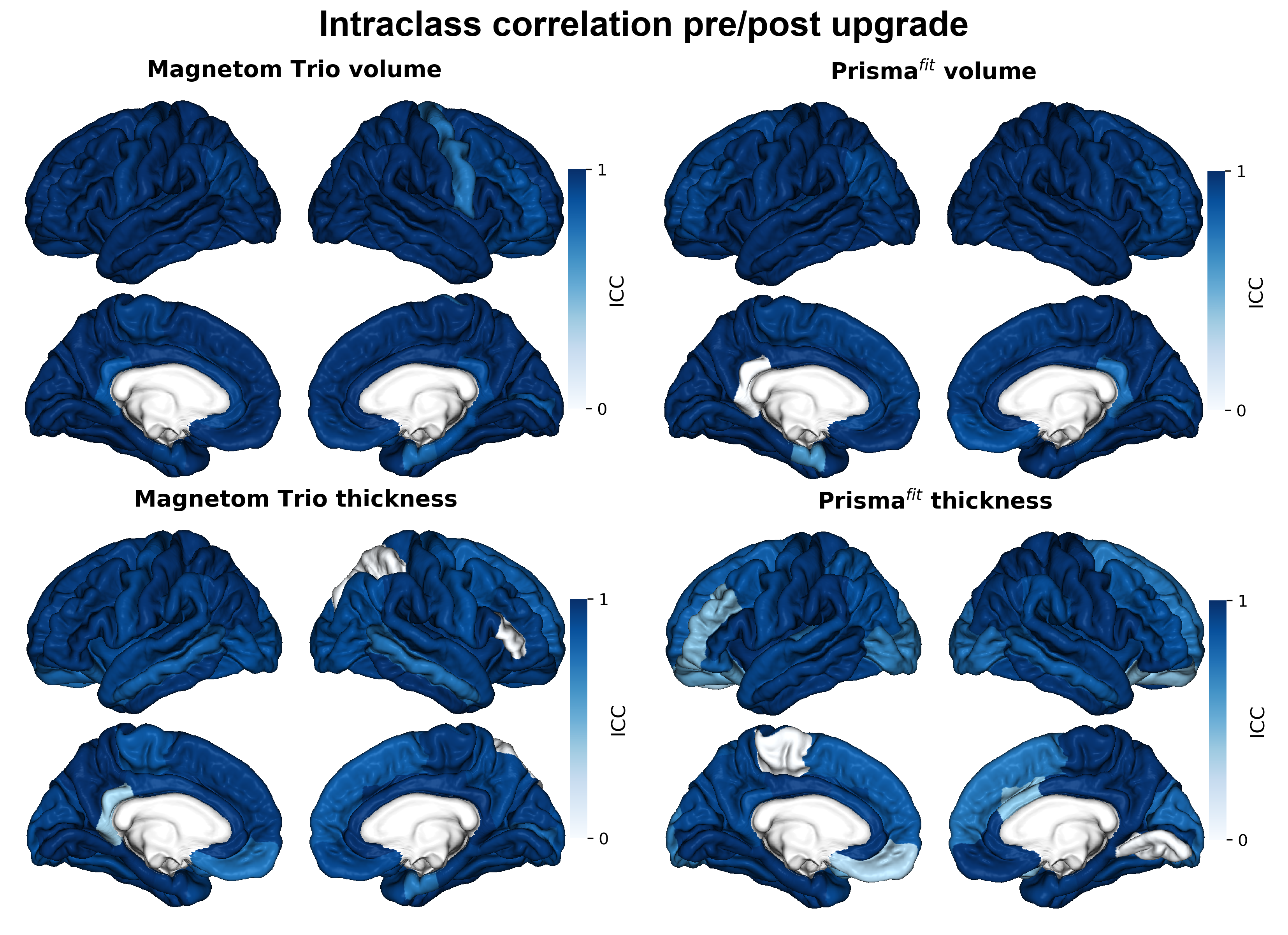

Supplement: Supplementary file 2 [file Image_1.TIFF]

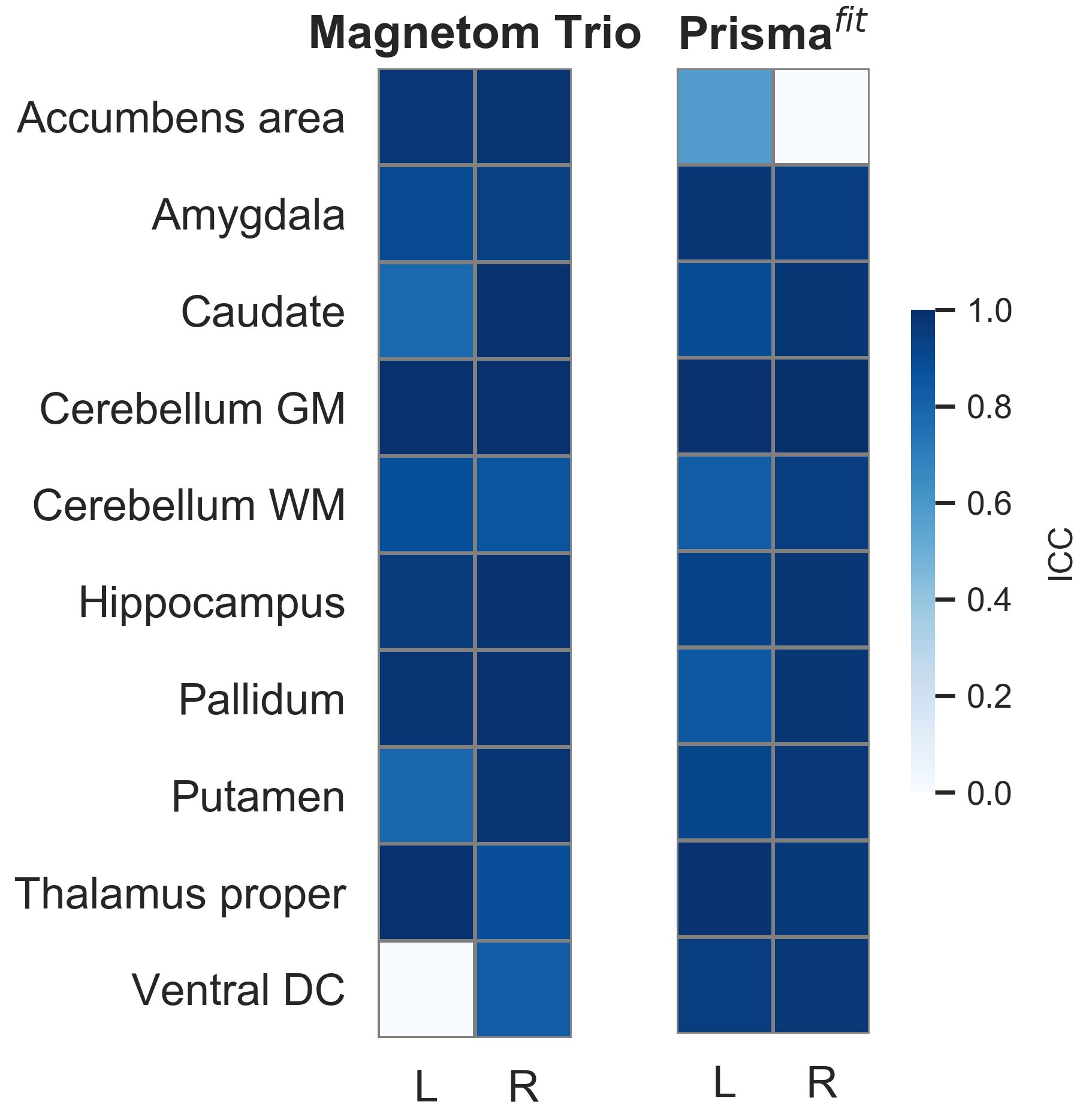

Supplement: Supplementary file 3 [file Image_2.TIFF]
